# Metabolic Interactions between Bacteria and Fungi in Commensal Oral Biofilms

**DOI:** 10.3390/jof3030040

**Published:** 2017-07-14

**Authors:** Marloes Lof, Marleen M. Janus, Bastiaan P. Krom

**Affiliations:** Department of Preventive Dentistry, Academic Centre for Dentistry Amsterdam (ACTA), Vrije Universiteit Amsterdam and the University of Amsterdam, Amsterdam, 1081 LA, The Netherlands; m3.lof@student.vu.nl (M.L.); marleenjanus@gmail.com (M.M.J.)

**Keywords:** oral biofilms, bacterium-fungus interactions, healthy oral cavity, oxygen gradients

## Abstract

Oral health is more than just the absence of disease. The key to oral health is a diverse microbiome in an ecological balance. The oral microbiota is one of the most complex and diverse microbial communities in the human body. To maintain oral health, balance between the human host and the intrinsic microorganisms is essential. The healthy oral cavity is represented by a great microbial diversity, including both bacteria and fungi. The bacterial microbiome is very well studied. In contrast, fungi inhabiting the oral cavity are often overlooked. All microbial species in the oral cavity form communities which establish a variety of micro-niches and inter- and intra-species interactions. These interactions can be classified into three main groups: physical, chemical and metabolic interactions. Different metabolic interactions are reviewed in this report, among which are the metabolism of sugars, carbon, lactate and oxygen. This review set out with the aim of assessing the importance of metabolic interactions between fungi and bacteria in the healthy oral cavity.

## 1. Introduction

### 1.1. Importance of Oral Health

People are aware that good oral health is very important. Consequently, daily brushing and flossing are taught to us from a very early age to keep our teeth as clean as possible. However, not everyone is aware that good oral health is more than pretty white teeth.

### 1.2. Oral Health Is Much More than Healthy Teeth

Good oral health also includes the gums and their supporting tissues, palate, lining of the mouth and throat, tongue, lips, salivary glands, chewing muscles, nerves, and the bones of the upper and lower jaws. Moreover, oral health is known to be integral to the rest of the body, which makes it a critical component of general health [1]. In addition, recent research has indicated that the oral microbiota plays an important role in regulation of blood pressure. Our blood pressure is regulated by a small diffusible molecule: NO (nitric oxide). Dietary components with high nitrate content, such as spinach and other green vegetables, are able to lower the blood pressure [2]. Nitrate (NO_3_) is converted to nitrite (NO_2_) and subsequently to nitric oxide (NO). We humans have enzymes capable of converting NO_2_ to NO, but we lack enzymes that convert NO_3_ to NO_2_. Upon digestion of dietary NO_3_, our body feeds back the NO_3_ to the oral cavity through the salivary glands. In the oral cavity bacteria convert the NO_3_ to NO_2_ that is taken up by the host. Oral disinfection using chlorhexidine prior to the consumption of dietary NO_3_ prevented the conversion to NO_2_ and subsequent lowering of the blood pressure [3]. Thus, the oral bacteria are truly important for a healthy physiology.

Oral health as such is more than just the absence of disease. The key to oral health is a diverse microbiome in an ecological balance that practices commensalism within itself and mutualism with the host [4]. This has also been indicated in the microbiome of children who suffer from severe dental caries. Caries-associated taxa included *Granulicatella elegans*, *Veillonella* spp., *S. mutans* and *Bifidobacteriaceae* spp. Species associated with caries-free children included among others *Capnocytophaga gingivalis*, *Abiotrophia defectiva*, *Streptococcus sanguinis* and *Streptococcus cristatus*. These microbiomes are less diverse when compared to the microbiome of children with good oral health [5].

### 1.3. Oral Microbiota Is Complex

The oral microbiota is one of the most complex and diverse microbial communities in the human body [6]. To maintain oral health, ecological balance between the human host and the intrinsic microorganisms is essential [7]. The oral cavity consists of a humid, nutrition-rich environment in which many microbes can thrive and form biofilms [8]. These oral biofilms (or dental plaques, when attached to tooth surfaces) were first observed in the seventeenth century by Antoni van Leeuwenhoek [9], and are involved in the formation of caries and periodontal disease [7]. Changes in environmental conditions, e.g. changes in dietary sugar intake, hormonal changes, smoking, etc., can lead to long-term changes in microbial ecology which can lead to tooth decay or inflammation of the soft tissue in the mouth. This ecological plaque hypothesis was posed by Marsh in 1994. He proposed the now widely accepted idea that the resident plaque microflora can shift to a disease-associated species composition by a change in a key environmental factor [10].

The healthy oral cavity is represented by a great diversity of bacteria and fungi. Much is known about the bacterial microbiome; it may harbor over 800–1000 different oral bacterial taxa [11]. This microbiome can be classified into the core microbiome, which is shared among all humans, and the variable microbiome, which is different between individuals [12]. The core microbiome contains predominant species that exist under healthy conditions. The variable microbiome differs per individual and has adapted to its lifestyle and phenotypic and genotypic determinants. Although the microbiome differs between individuals, the microbiome per individual seems to be consistently the same in time [13]. In contrast with the bacterial microbiome, fungi inhabiting the oral cavity are often overlooked [14]. This review focuses on the different levels of interactions between oral bacteria and fungi, with most emphasis on metabolic interactions.

### 1.4. Bacteria

**Figure 1:**
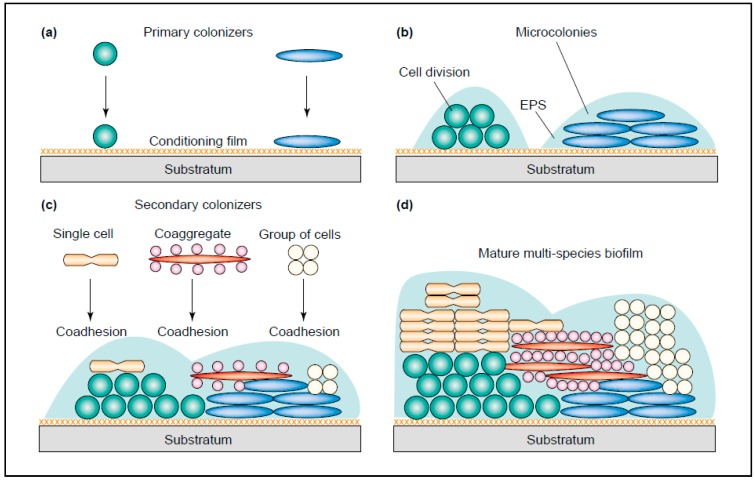
The development of a multi-species biofilm (plaque). (**a**) Early colonizers bind to the conditioning film (pellicle) (**b**) The early colonizers start to grow and divide and form microcolonies. The production of EPS starts (**c**) Co-adhesion of single cells, groups of cells and co-aggregates occur (**d**) Maturation of the complete plaque (Image taken from: [19] with permission).

Bacteria in the oral cavity do not exist as isolated cells, but grow and survive in organized communities. These plaques can be formed at phase interfaces, for example the solid–liquid interphase in the mouth [8]. The development of a new plaque starts with the coverage of the tooth surface with a protein film (pellicle). This process is very fast and takes only minutes to form after eruption of a new tooth or after elimination of an existing plaque [15]. The pellicle exists of several molecules derived from the host, containing mucins, proteins and agglutinins, which are recognized by a number of bacteria. These “early colonizers” can bind to the pellicle-covered surface of the tooth and include mostly streptococci and several gram-positive rods (Rickard, 2003). Most frequently, *Streptococcus* spp., *Actinomyces* spp., *Capnocytophaga* spp., *Eikenella* spp., *Haemophilus* spp., *Prevotella* spp., *Propionibacterium* spp. and *Veillonella* spp. are found [16]. Successively, these early colonizers are used for the “late colonizers” to adhere to, including, *Actinobacillus* spp., *Prevotella* spp., *Eubacterium* spp., *Porphorymonas* spp. and *Treponema* spp. (Figure 1). Between pairs of early colonizers, co-aggregation is common, where this is rare between early and late colonizers. Additionally, several “bridging” bacteria have been found. These bacteria, such as *Fusobacterium nucleatum*, are thought to form a bridge between early and late colonizers, since they are shown to co-aggregate with both species. *Fusobacterium nucleatum* is also able to bind to many host-derived molecules found in the pellicle [17]. As both the early and late colonizers grow and divide, many will start to express a biofilm phenotype and start to secrete extracellular polymeric substances (EPS), including polypeptides, carbohydrates and nucleic acids. These EPS form the majority of the mature biofilm structure which affords physical stability and protection from the flow of saliva and other forces in the oral cavity [18].

Dental plaques often consist of at least 800 bacterial species and can develop under a range of different conditions [4]. Under healthy conditions, these plaques play an important role in the defense mechanisms in the host. However, dental plaques are also associated with dental caries and periodontitis. Dental plaque bacteria are frequently faced with factors that challenge a health-compatible state, including exposure to high-sugar-content foods and tobacco smoke [8].

### 1.5. Diseases of the Mouth

Diseases we will focus on in this review are dental caries, periodontal disease (i.e., gingivitis and periodontitis) and halitosis since these diseases are the most prevalent in adults. Once it was thought that dental caries was one simple disease which was caused by a single bacterium, *Streptococcus mutans* [20]. Therefore, treatments were solely targeted at this species. More recently, it has been found that dental caries is a far more complex disease, in which the hard tissue of the tooth is demineralized in response to a multifaceted microbial challenge [4]. A direct cause of dental caries turned out to be exposure of the teeth to carbohydrate, after which several acidogenic and aciduric bacterial species can increase in number and form a cariogenic plaque [21]. Below the critical pH of 5.5, demineralization of the teeth occurs while above the critical pH remineralization of the teeth occurs. The carbohydrate is metabolized by acidogenic bacteria and this results in a lower pH in the cariogenic plaque (pH < 5). This attacks the enamel and eventually causes cavitation to occur [22]. Besides *S. mutans*, other bacterial species can be found in the plaque, such as other low-pH streptococci (*S. oralis*, *S. mitis*, and *S. anginosus*), *Rothia*, *Actinomyces*, *lactobacilli* and *Bifidobacterium* species [4].

Periodontal disease, including gingivitis and periodontitis, is inflammation of the gum tissue lining the teeth. Gingivitis is represented by mild inflammation and swelling of the gums with bleeding upon touch. Generally, gingivitis is a reversible inflammation which is resolved upon reinstallation of proper oral hygiene. However, when not treated properly, gingivitis can progress to periodontitis, which involves the irreversible degradation of the alveolar bone around the teeth, ultimately leading to tooth-loss.

Most cases of gingivitis are caused by accumulation of dental plaque. Within these biofilms, microbes produce degradative enzymes, e.g. proteases and toxins (lipopolysaccharide (LPS) and lipoteichoic acid (LTA)), which can induce an inflammatory response in the gum tissue. Early dental plaque in healthy individuals consist of a bacterial community dominated by Gram-positive cocci and rods. As time proceeds oral health deteriorates due to changes in the microbial communities consisting of more Gram-negative rods, fusiforms, filaments, spirilla and spirochetes. Specific taxa associated with gingivitis are *Fusobacterium nucleatum* subsp. *polymorphum*, *Lachnospiraceae* (G-2) species HOT100, *Lautropia* sp. HOTA94, and *Prevotella oulorum*. Bacteria of the red-complex, such as *Aggregatibacter actinomycetemcomitans*, fulfil; Koch’s postulates as the predominant etiological agent of periodontitis. Although periodontitis is a complex disease, there are a number of bacteria associated with the majority of disease cases [23].

Halitosis (bad breath) is the third major reason, after dental caries and periodontal disease, for many people to seek dental care. Approximately 90% of halitosis cases are caused by various oral conditions. The minority of the cases is caused by systemic diseases, e.g. gastrointestinal disorders, hepatic diseases and diabetes [24]. Halitosis with oral origin is caused by the overgrowth of proteolytic anaerobic bacteria which reside on the surface of the tongue [25]. Volatile sulfur compounds (VSCs) are the primary reason for the foul smell of halitosis. VSCs are a by-product of microbial metabolic degradation and include hydrogen sulfide (H_2_S), methyl mercaptan (CH_3_SH) and dimethyl sulfide ((CH_3_)_2_S). The most common bacterial species known to produce VSCs are *Porphyromonas*, *Prevotella*, *Actinobacillus* and *Fusobacterium* [26].

The oral cavity affords a doorway to the rest of the body, through the alimentary canal, the gingival crevice or through the wounds in mucosal surfaces that arise after brushing or eating, which may lead to short term bacteremia [27]. Additionally, oral bacteria can often be isolated from the blood of patients after procedures such as tooth extraction. Many of these isolated bacteria have been shown to cause endocarditis [28]. Research has demonstrated associations between oral bacteria and systemic diseases, e.g., coronary disease, and a higher risk of preterm, low birth weight babies [29,30].

Oral health is integral to general health. Since it is a critical component of general health, one cannot be healthy without good oral health. The majority of bacteria living in the oral cavity do so in symbiosis of the host. The normal oral biofilm has mostly a beneficial effect since its constant presence provides a resistance against possible pathogenic microorganisms. This is also shown by the change in populations of predominant bacteria in a plaque from a healthy state to a disease state [31].

### 1.6. Fungi

Fungi are ubiquitous organisms that colonize humans, animals, fruits, vegetables and other plant material. Since they are omnipresent and abundant in the environment, all plants and animals have co-evolved with them. Living with fungi can be both beneficial and harmful. An example of a beneficial relationship with humans are members of the *Nitrososphaera* genus. These fungi can be found in the gut and are able to oxidize ammonia and degrade urea. Seemingly, this also feeds nitrogen to the gut microbial community [32]. On the other hand, some fungal species can cause disease. For example, the fungus *Cryptococcus neoformans* can cause infection, then distribute to the central nervous system and cause life-threatening meningoencephalitis in immunocompromised individuals. Nevertheless, *Cryptococcus* spp. are common in the environment and commonly inhaled without causing disease since the immune system is usually able to dispose of it [33]. The fungi *Histoplasma capsulatum* and *Coccidiodes immitis* can occasionally cause disseminated infections which are lethal if not treated specifically [34]. Consequently, humans have developed specific mechanisms to both live with fungi and protect itself against fungal disease. Many fungal species are normally tolerated to inhabit different surfaces of the human body. Of the more than 100,000 fungal species, which are widespread, only few are known to cause diseases in humans [33].

Since fungi have to survive as inhabitants of environmental niches; they must be able to adapt to constantly changing parameters. These niches are highly dynamic and can be composed of both biotic and abiotic factors [35]. One of those niches is the oral cavity.

In the oral cavity of healthy individuals, over 100 fungal species have been identified [36]. *Candida* species have been found to be the most frequent in healthy individuals, and could be isolated from 75% of the individuals, followed by *Cladosporium*, *Aureobasidium*, *Saccharomycetales*, *Aspergillus*, *Fusarium* and *Cryptococcus*. The distribution of fungal species varies greatly between different individuals. Importantly, in 20% of the individuals, the four most common opportunistic pathogenic fungi were identified as *Candida*, *Aspergillus*, *Fusarium* and *Cryptococcus*. The most abundant *Candida* species found were *C. albicans* (in 40% of the subjects), followed by *C. parapsilosis* (15%), *C. tropicalis* (15%), *C. khmerensis* (5%) and *C. metapsilosis* (5%) [36].

Three years later, another study was conducted using an improved cultivation method to characterize the oral fungal microbiota in healthy participants [37]. It was shown that oral fungal colonization was found in all participants, even though there was a large species variability between individuals. Nevertheless, the fungal taxa diversity and concentrations were largely consistent in each participant. These results were consistent with the previously mentioned results from Ghannoum et al. [36].

*Candida* species have been found to be the most important and most prevalent colonizing fungi in the oral cavity. Even though they are prevalent in the majority of healthy individuals, they are associated with oral diseases such as oral candidiasis. However, only a select group of mostly immunocompromised individuals develop candidiasis. In 80% of these cases, *C. albicans* is responsible for the lesions [38]. Another well-known example of fungal disease targeting the immunocompromised would include *Aspergillus* spp.. Humans often come into contact with *Aspergillus* spp. The spores of this fungus are common in the environment and frequently inhaled and cleared in immunocompetent people [33]. More recently, an *A. niger* variant was isolated from the oral cavity of a young woman with underlying disease (B.P. Krom, unpublished data). *Cryptococcus* is also often found in the healthy oral cavity [33]. Contrary to *Candida* spp., however, these two species are generally not thought to be a commensal species in the oral or naso-pharyngeal microbiome.

## 2. Interactions between Fungi and Bacteria Might Be Important for Maintenance of the Healthy Oral Ecology

**Figure 2:**
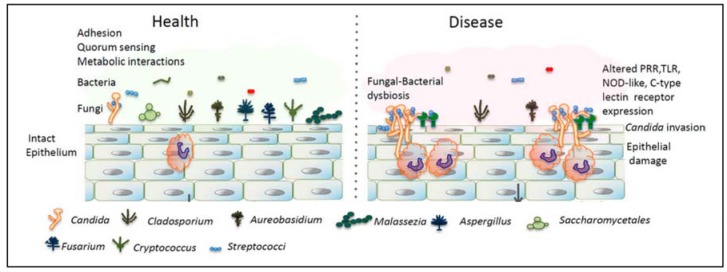
Differences in the mycobiota in the oral cavity in health and disease. A dysbiotic state of C*andida* overgrowth causes epithelial damage. Adapted from [39] with permission.

Microbial species in the oral cavity form communities that establish a variety of micro-niches, and inter- and intra-species interactions. For fungi to survive and grow in the oral cavity, they should be in a symbiotic relationship with the residential bacterial microbiota and the host. Since *Candida* species are the easiest to isolate, and are found to be the most highly abundant, most (culture) studies considering the effect of bacteria on fungi have been done on this genus. For example, Figure 2 shows the difference between the microbiota of the healthy oral cavity and the oral cavity in a state of imbalance. In the latter, *Candida* species have overgrown and a disease state arises. Several commensal bacteria are now able to interact with *Candida* spp. which results in an increased inflammatory response. This response can result in epithelial damage [39].

The interactions between the different microbial species can be classified into three main groups: physical, chemical and metabolic interactions. Since the emphasis of this paper will be on the metabolic interactions between fungi and bacteria, physical interactions will only be mentioned briefly. Although quorum sensing is often referred to as a form of chemical interaction, it could also be seen as a form of metabolic interaction.

Physical interactions, both non-specific (electrostatic and hydrophobic) and specific (protein–protein) interactions, occur among different types of bacterial cells with almost all known oral bacterial strains. This process is called co-aggregation when this happens between suspended cells. When suspended cells attach to cells, which are already in a biofilm, this process is called co-adhesion. Nevertheless, the mechanisms of these two processes are the same and may overlap since co-aggregates are able to adhere unto the biofilm. These interactions make sure that bacterial cells are closely together and enable them to communicate through signaling and genetic exchange [40]. Physical interactions can both happen between bacteria, and between fungi and bacteria. For example, *Streptococcus* sp. and *C. albicans* can form intimate corn-cobb-like structures in the supragingival plaque [41]. The absolute number of fungal species in the oral cavity is much lower than the absolute number of bacterial species. Nevertheless, due to their greater cell size, fungi contribute to a larger extent to the total biomass. Additionally, since fungi are able to form thread-like filaments (hyphae); they are able to form a structural basis for fungal–bacterial plaques. In this way, fungi have a greater effect on the total oral microbiota than would be expected on their relative small number. This brought forward the hypothesis that fungal species are “keystone species” within the oral cavity [42].

**Figure 3:**
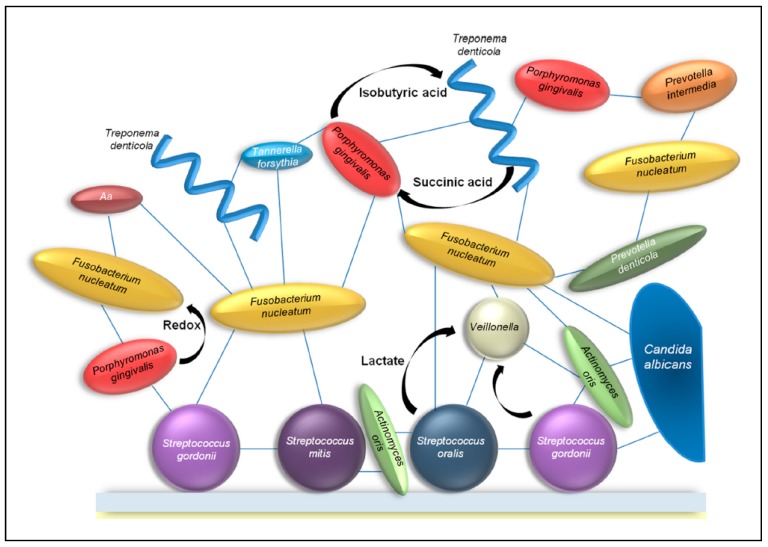
Example of several interactions in the oral biofilm. Each blue line indicates a physical interaction between different bacteria and fungi. The black arrows indicate the release of metabolic factors by one bacterium which is used by other bacteria. (Aa = *Aggregatibacter actinomycetemcomitans*). Image taken from: [43] with permission.

The recognition and co-adhesion between different species of bacteria can give an advantage to all participants. When growing in a community, bacteria can benefit from the metabolic activity of nearby bacteria, and gain access to nutrients that would not be available to planktonic bacteria. Therefore, bacteria often co-localize with others that are metabolically compatible [43]. To become incorporated in oral microbial communities, bacteria often rely on metabolic cooperation of other bacteria and fungi. This results in a web of metabolic exchanges between bacteria and fungi (Figure 3). Within a plaque, different kinds of metabolic interactions can occur. Competitive metabolic interactions occur when two species consume the same resource. Cooperative metabolic interactions happen when metabolites produced by one species are consumed by another species.

As mentioned before, quorum sensing could also be seen as a form of metabolic interaction. This process regulates numerous important biological processes in bacteria by releasing signal molecules. These molecules, termed autoinducers, increase in concentration when bacterial density is high. Detection of autoinducers leads, after a certain threshold is exceeded, to changes in gene expression in the bacteria and subsequently to a response of the entire population [44].

Jarosz et al. have investigated the communication between the fungus *C. albicans* and cariogenic bacteria *S. mutans*. They found that *S. mutans* was able to inhibit *C. albicans* germ tube formation in co-cultures. Since they were physically separated it was not due to some physical interaction. Instead, it was found that a specific bacterial quorum sensing molecule was responsible for this inhibition. This molecule, competence-stimulating peptide (CSP), is produced by *S. mutans* during early stages of growth [45].

Another example is the Gram-negative bacterium *A. actinomycetemcomitans*. This opportunistic bacterium is able to cause aggressive periodontal disease in some cases, but is generally a commensal of the oral cavity. It produces autoinducer-2 (AI-2), which is an example of a more generic quorum sensing molecule. *A. actinomycetemcomitans* is known to inhibit *Candida* biofilm formation by the production of AI-2 [46]. AI-2 is also secreted by *Streptococcus* spp. However, different bacterial species produce somewhat different AI-2 derivatives, meaning that results from one species’ autoinducer are not representative for another’s. Consequently, the role of AI-2 in the interaction between *S. mutans* and *C. albicans* has still to be studied in the future [45].

A third example of a quorum signalling bacterium is the Gram-negative *Pseudomonas aeruginosa* which forms biofilms on *C. albicans*. *P. aeruginosa* produces the quorum sensing molecule 3-oxo-C_12_ homoserine lactone (3OC_12_HSL) which has shown to suppress *C. albicans* filamentation in vitro. However, *P. aeruginosa* neither attaches to nor kills *C. albicans* yeast-form cells. Therefore, 3OC_12_HSL can lead to fungal growth, since the resistant yeast can proliferate despite the presence of filament-inducing conditions [47].

Some bacterial species have evolved mechanisms to inhibit growth and attachment of competing bacteria. For example, streptococci produce the small metabolic molecule hydrogen peroxide (H_2_O_2_) to actively reduce the growth of other bacteria [43]. In this way, *S. gordonii* is also able to influence *C. albicans*. Hydrogen peroxide produced by this bacterium influences morphogenesis and farnesol production of *C. albicans*. Farnesol acts as a QS molecule and represses the formation of hyphae. Since hyphal formation is linked to invasion of the epithelial tissue, this could be a way in which bacteria are able to control *C. albicans* pathogenesis in the oral cavity [48].

Most known metabolic interactions that are not related to quorum sensing, are related to the essential and highly conserved central carbon metabolism. An example of direct metabolic interactions between bacteria in the oral cavity is the growth of streptococci, which leads to the production of lactate. Lactate is a preferred substrate for *Veillonella atypica*. The Gram-negative anaerobic *Veillonella* spp. are often found in dental plaques in high abundance [49] and are known to convert lactate to weaker acids. Furthermore, it is shown that when *Streptococcus gordonii* and *V. atypica* are grown together, a diffusible signal causes the amyB gene to be upregulated by the *S. gordonii* amylase gene. This increased amylase activity causes more lactic acid to be produced, causing even more favorable circumstances for *V. atypica* [50]. The consumption of lactate by *V. atypica* removes the end product of the glycolysis and causes the glycolysis rate in the streptococci to increase [43]. In these ways, *Veillonella* spp. are able to influence the cariogenic potential of the dental plaque. Besides *Veillonella* species, several strains of *Neisseria, Corynebacterium,* and *Eubacterium* are also able to metabolize lactate and convert it into weaker acids [51].

Not only bacteria, but also most fungi have extremely versatile metabolisms which allow them to quickly adapt to changing environmental conditions. Several crucial cellular events, such as morphogenesis, are often accompanied by changes in pathways including catabolic and anabolic biochemical reactions. Several polymorphic fungi, such as *C. albicans*, are able to grow in different morphological forms. Depending on environmental conditions, they may switch from a filamentous form to a yeast form. The ability to switch between these forms is considered a crucial virulence factor of these fungi. The filamentous form is notorious for its ability to be invasive through the host epithelial surface and its high resistance to phagocytosis by macrophages. On the other hand, the yeast form is able to disseminate through the bloodstream and adhere to different endothelial surfaces [52]. Since changes to different morphological forms have an important influence on the virulence of the fungi and these changes are accompanied by changes in metabolic pathways, it is important to understand these changes in central carbon metabolism.

Additionally, understanding of fungal central carbon metabolism is important as it potentiates a plethora of metabolic interactions between bacteria and fungi. It is likely that these interactions are key in the establishment of a healthy oral ecology. For example, *C. albicans* is also able to utilize lactate besides sucrose and glucose. Since *C. albicans* is often found in the presence of *S. mutans* [53], it is likely that the growth of *C. albicans* is encouraged by the lactate produced by *S. mutans* [54]. Thus, the utilization of lactate by *C. albicans* should cause a less cariogenic environment in the same way as *Veillonella* spp.

Moreover, a study done by Willems et al. showed that the presence of *C. albicans* in a biofilm which also includes *S. mutans* decreases the cariogenic potential of the plaque. The studied dual-species biofilm showed a decrease in acidity over time in which the external pH in co-cultures remained above the critical 5.3–5.5. This decrease in acidity could result from *C. albicans* metabolizing the lactate produced by *S. mutans*, or by active modulation of extracellular pH by *C. albicans*. Several studies indicate that *C. albicans* has evolved in more ways to modulate the pH of the environment. For example, *C. albicans* is able to generate ammonia when catabolizing amino acids. Hereby it is able to raise the extracellular pH and subsequently promote hyphal morphogenesis [55]. Moreover, these researchers showed a comparable process in which *C. albicans* is able to neutralize an acidic extracellular environment by using carboxylic acids as carbon source, such as α-ketoglutarate (αKG), pyruvate, or lactate. In this way, *C. albicans* does not excrete ammonia and does not form hyphae, which is an important difference from the process which is driven by amino acids [56]. Since extracellular alkalization has been reported in more fungal species [57] this process could be an important factor that helps the survival of fungi in the host. This prevents the acidification of the oral cavity by preventing accumulation of acids. Other bacteria, to which an acidic environment is toxic, are dependent on these neutralizing activities for their survival. Acid removal and modulation of extracellular pH thus counteracts a microbial switch towards a cariogenic community. Therefore, extracellular alkalization by fungi might be an important contribution to the oral health.

Metabolic dependency can also occur in a less direct way. Indirect metabolic dependency can occur after the removal of certain metabolites by one species which are toxic for other species, allowing these to grow. An example of this are particular anaerobic bacteria which are dependent on the reduction of local oxygen levels by the rapid oxygen consumption of aerobic bacteria [58]. Consequently, it could be that these interactions are important in the establishment and the maintenance of the healthy diverse oral ecology.

*C. albicans* is able to grow both aerobically and anaerobically. Additionally, it is able to remove the limited oxygen available in its surroundings [59,60,61]. Hereby *C. albicans* is responsible for the formation of a hypoxic microenvironment. By reducing the oxygen tension, anaerobic bacteria are able to live within the plaque. Anaerobic bacteria are even able to live when cultured in ambient oxic conditions, even though this kind of environment is usually lethal to anaerobic bacteria [59]. Since both aerobic and anaerobic bacteria are able to live within a plaque containing oxygen consuming *C. albicans,* a higher biodiversity is possible. For example, the survival and the colonization of the bacterium *S. gordonii* is promoted by *C. albicans* [54] and likewise growth of *C. perfringens* and *B. fragilis* under aerobic conditions is also facilitated by *C. albicans*. Furthermore, a recent study found that the bacterium *C. difficile* is able to survive in an aerobic environment when in the presence of *C. albicans*, whereas these conditions would be toxic in the absence of *C. albicans* [61]. A recent in vitro study showed that in oral biofilms presence of *C. albicans* does indeed lead to the presence of strict anaerobes within aerobically cultured dental plaque [60]. Consequently, a higher diversity was found in dental plaque containing *C. albicans* compared to dental plaque without. This way, *C. albicans,* but possibly also other fungi, contribute to a more diverse microenvironment, commonly regarded as a healthier ecosystem, by the depletion of oxygen in the oral cavity.

These diverse microenvironments may change cellular pathways in ways previously undiscovered due to the binary nature of most interaction studies. A co-culture containing both bacteria and fungi is able to induce a change in the expression of genes in *C. albicans*. For example, a multi-species study showed that when *C. albicans* was co-cultured with the bacteria *K. pneumoniae*, *E. coli*, and *E. faecalis*, several genes changed their expression pattern. In particular, the WOR1 gene was strongly upregulated by the presence of these bacteria. This gene causes the change of *C. albicans* from a white phenotype to an opaque phenotype [59] even though *C. albicans* normally expresses the white phenotype at the physiological temperature of 37 °C [62]. This phenotypical switch facilitates the mating process in *C. albicans* [63]. Thus, the presence of bacteria may be able to influence the mating of *C. albicans*.

### 2.1. Volatile Sulfur Compounds

As mentioned previously, halitosis is caused by the production of VSCs by mainly proteolytic anaerobic bacteria. Sulfur is an essential element for many organisms in the human host. Its metabolism by fungi might provide a beneficial effect against halitosis. A study by Amich et al. showed that the fungus *Aspergillus fumigatus* is able to take up VSCs produced from methionine catabolism and to use them as S-source [64]. Additionally, the fungus *C. glabrata* is able to utilize the sulfur-containing molecule glutathione [65]. Furthermore, several filamentous fungi, such as *A. nidulans* and *Neurospora crassa*, and the yeast *Neurospora crassa* have the ability to obtain sulfur from the environment [66]. Possibly, this means that halitosis could be countered by the metabolism of VSCs by fungal species. This implies that if this mechanism is activated in the oral cavity, the metabolism of fungi would provide a beneficial effect against halitosis.

## 3. Discussion and Conclusions

This review set out with the aim of assessing the importance of metabolic interactions between fungi and bacteria in the healthy oral cavity. As mentioned, there are several ways in which fungi and bacteria can interact, both physically and metabolically. Different metabolic interactions are reviewed in this report, among which are the metabolism of carbon, lactate and oxygen. These findings further support the idea of the important role of metabolic interactions between fungi and bacteria in the healthy oral cavity.

Remarkably, most research in this field has been done with one specific fungus, *Candida albicans*. To develop a full picture of the role of fungi in the healthy oral cavity, additional studies are needed that will focus on metabolic interactions between bacteria and other fungi present in the oral cavity, such as *Cladosporium*, *Aureobasidium*, *Saccharomycetales*, *Aspergillus*, *Fusarium* and *Cryptococcus*.

Moreover, the complete oral microbial interactome is not complete without detailed information about the fungi in the oral cavity. Fungi have often only been studied in relation to disease, which gives an overall wrong impression about these microorganisms. Therefore, the beneficial role fungi may have been overlooked.

We envision that in the following years it will turn out that fungi, not just yeasts such as *C. albicans*, have a beneficial role in the care of the oral ecosystem and, subsequently, are important for oral health. We recommend more research to be done in this field, which will undoubtedly lead to a more extensive metabolic map including fungi as well as bacteria.
